# The contribution of arachidonate 15-lipoxygenase in tissue macrophages to adipose tissue remodeling

**DOI:** 10.1038/cddis.2016.190

**Published:** 2016-06-30

**Authors:** H-J Kwon, S-N Kim, Y-A Kim, Y-H Lee

**Affiliations:** 1 College of Pharmacy, Yonsei University, Incheon 21983, South Korea

## Abstract

Cellular plasticity in adipose tissue involves adipocyte death, its clearance, and *de novo* adipogenesis, enabling homeostatic turnover and adaptation to metabolic challenges; however, mechanisms regulating these serial events are not fully understood. The present study investigated the roles of arachidonate 15-lipoxygenase (Alox15) in the clearance of dying adipocytes by adipose tissue macrophages. First, upregulation of Alox15 expression and apoptotic adipocyte death in gonadal white adipose tissue (gWAT) were characterized during adipose tissue remodeling induced by *β*3-adrenergic receptor stimulation. Next, an *in vitro* reconstruction of adipose tissue macrophages and apoptotic adipocytes recapitulated adipocyte clearance by macrophages and demonstrated that macrophages co-cultured with apoptotic adipocytes increased the expression of efferocytosis-related genes. Genetic deletion and pharmacological inhibition of Alox15 diminished the levels of adipocyte clearance by macrophages in a co-culture system. Gene expression profiling of macrophages isolated from gWAT of Alox15 knockout (KO) mice demonstrated distinct phenotypes, especially downregulation of genes involved in lipid uptake and metabolism compared to wild-type mice. Finally, *in vivo* *β*3-adrenergic stimulation in Alox15 KO mice failed to recruit crown-like structures, a macrophage network clearing dying adipocytes in gWAT. Consequently, in Alox15 KO mice, proliferation/differentiation of adipocyte progenitors and *β*3-adrenergic remodeling of gWAT were impaired compared to wild-type control mice. Collectively, our data established a pivotal role of Alox15 in the resolution of adipocyte death and in adipose tissue remodeling.

Macrophages exist in nearly all tissues as one of the major components of innate immunity, having a critical role in normal development and during tissue remodeling and repair.^1^ Although phagocytic ability to eliminate undesired pathogens is thought to be central to macrophage function, efferocytosis of apoptotic cells by macrophages is essential to tissue development, restoration, as well as resolution of inflammation.^2, 3^

Adipose tissue is a central metabolic organ that can store excess energy, mainly as triglycerides, and mobilize free fatty acids when energy is needed.^4, 5^ To perform its energy buffering function, adipose tissue is evolved to be highly plastic in terms of regulation of adipocyte cellularity.^4, 5^ During homeostatic maintenance, adipocytes turn over with tight regulation of *de novo* adipogenesis and cell death, and this process can be accelerated by energetic challenges.^5^ For instance, lipolysis by physiological (e.g., fasting) and pharmacological (e.g., *β*3-adrenergic receptor agonist) stimuli can induce adipocyte death defined by surrounding macrophages, which form a so-called crown-like structure (CLS) and proliferation/differentiation of adipocyte progenitors.^6, 7 and 8^ Importantly, pathological conditions such as inflamed hypertrophic adipose tissues induce adipocyte death, yet manifest unresolved chronic inflammation, indicated by CLS with giant multinucleated macrophages.^9, 10, 11 and 12^ This implies delayed clearance of dying adipocytes. Thus, mechanistic understanding of adipocyte death resolution is essential for healthy adipose tissue remodeling; however, mechanisms regulating these serial events are not fully understood.

Efferocytosis, that is, non-inflammatory removal of apoptotic cells, is distinct from the classic forms of phagocytosis, differing in recognition mechanisms and signaling pathways.^3^ Various lipid metabolites have an important role in the recognition and uptake of apoptotic cells.^2^ Particularly, arachidonate 15-lipoxygenase (Alox15) orchestrates non-inflammatory removal of apoptotic cells by generating oxidation products of phosphatidylethanolamine.^13, 14^ A previous study has shown that *β*3-adrenergic remodeling of white adipose tissue (WAT) recruits anti-inflammatory M2 macrophages with high expression levels of Alox15.^15^

In this study, the roles of Alox15 in the clearance of dying adipocytes were investigated by using an *in vitro* co-culture system and *β*3-adrenergic remodeling of adipose tissue in conjunction with Alox15 knockout (KO) mouse models. First, treatment with a *β*3-adrenergic receptor agonist, CL316,243 (CL), resulted in apoptotic cell death of adipocytes and increased Alox15 expression in macrophages. An *in vitro* co-culture study confirmed that macrophages efferocytosed dying adipocytes and had increased expression of Alox15 and efferocytosis-related genes. In this system, pharmacologic inhibition and genetic deletion of Alox15 diminished the levels of adipocyte clearance by macrophages. Gene expression profiling of macrophages isolated from gWAT of Alox15 KO mice demonstrated distinct phenotypes, notably, downregulation of gene related lipid uptake and metabolism compared to the wild-type (WT) mice. Finally, CL treatment failed to recruit M2 macrophages to form a network for clearing dying adipocytes in gWAT of Alox15 KO mice. Collectively, this study identified Alox15 activation in adipose tissue macrophages as a mechanism of non-inflammatory removal of dying adipocytes.

## Results

### *β*3-adrenergic stimulation induces apoptosis of adipocytes in gonadal WAT

**Figure 1:**
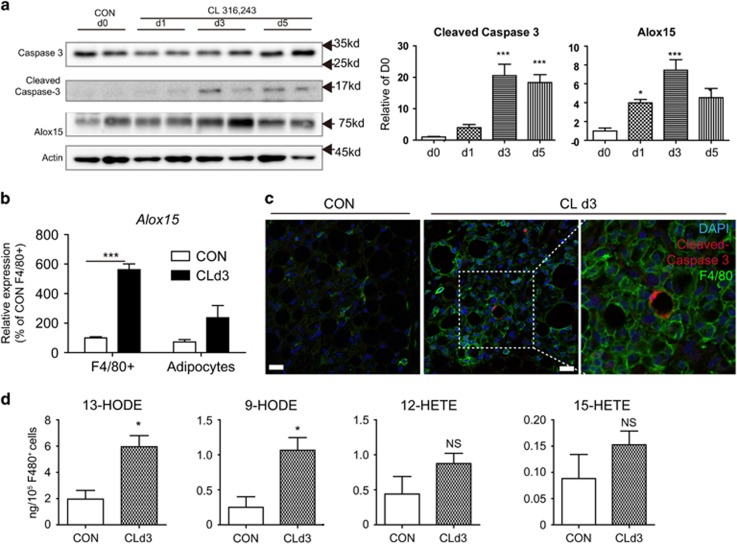
*β*3-adrenergic stimulation induces apoptosis of adipocytes in gonadal adipose tissue. (**a**) Immunoblot analysis and quantification of caspase-3, cleaved caspase-3 and Alox15 expression in gWAT from mice treated with CL up to 5 days. *β*-actin was used as a loading control. Error bars indicated S.E.M. of four individual experiments. (*P<0.05, ***P<0.001). (**b**) qPCR analysis of Alox15 expression in F4/80+ macrophages and adipocytes from of mice treated with CL for 3 days and untreated controls. Error bars indicated S.E.M. of three individual experiments (***P<0.001). (**c**) Immunohistochemistry of cleaved caspase-3 and F4/80 in paraffin sections of gWAT of mice treated with CL for 3 days and untreated controls. Nuclei were counterstained with DAPI. Bars=20 *μ*m. (**d**) Analysis of Alox15-dependent lipid metabolites in isolated F4/80+ macrophages from gWAT of mice treated with CL for 3 days and untreated controls . Error bars indicated S.E.M. of three individual experiments per condition. Three biological replicates of pooled tissues from four mice were analyzed (*P<0.05, N.S. = non-significant)

Our previous work established that *β*3-adrenergic stimulation can recruit anti-inflammatory macrophages that surround lipid remnants of dying adipocytes in gWAT;^6^ however, adipocyte death had not been mechanistically characterized in this model. To determine whether *in vivo* activation of lipolysis can induce apoptotic cell death in adipose tissue, mice were treated with CL for up to 5 days. Caspase-3 activation was used as an indicator of apoptosis. The detected amount of cleaved caspase-3 increased during the course of CL treatment, peaking at 3 days (Figure 1a). Along with increased levels of cleaved caspase-3, Alox15 expression was upregulated and it also peaked at day 3 of CL treatment,^6^ as shown in Figure 1a. To determine which cell types express Alox15, F4/80+ cells and adipocytes were isolated from gWAT of untreated control mice and mice treated with CL for 3 days. Although the expression levels of Alox15 were similar between F4/80+ macrophages and adipocytes under control conditions, CL treatment significantly increased Alox15 expression in F4/80+ macrophages, but not in adipocytes (Figure 1b). Double staining on histological paraffin sections for cleaved caspase-3 and a macrophage marker, F4/80 indicated that apoptotic adipocytes were surrounded by macrophages in gWAT after 3 days of CL treatment (Figure 1c). Next, we performed LC–MS/MS lipidomic analysis of isolated F4/80+ macrophages from gWAT of CL-treated mice and untreated controls to determine the levels of Alox15 products. Consistent with our previous report,^15^ the PPAR*γ* ligands 9-hydroxyoctadecadienoic acid (HODE) and 13-HODE, were significantly upregulated in isolated adipose tissue macrophages after CL treatment (Figure 1d). In contrast, the levels of pro-inflammatory lipid metabolites such as 12-hydroxyeicosatetraenoic acid (HETE)^16, 17^ and 15-HETE, were not significantly changed in F4/80+ macrophages after CL treatment (Figure 1d).

### Alox15 is required for efferocytosis of apoptotic adipocytes by macrophages *in vitro*

**Figure 2:**
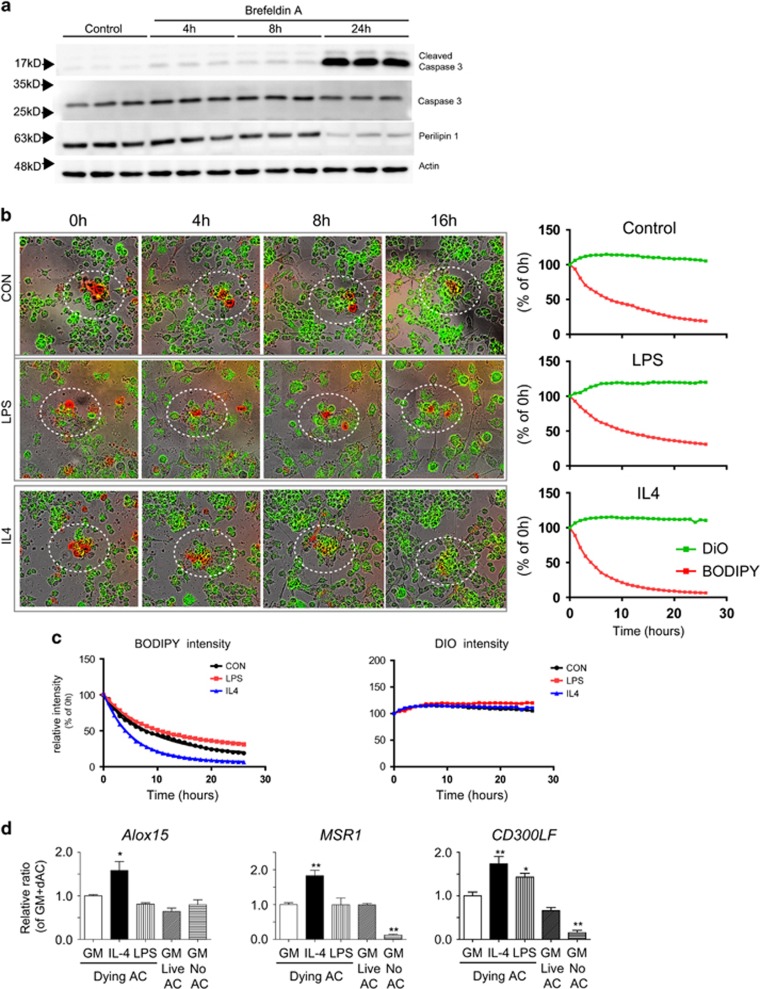
*In vitro* co-culture recapitulates adipocyte clearance by M2-like adipose tissue macrophages. (**a**) Immunoblot analysis of caspase-3 activation and perilipin1 expression in differentiated adipocytes from C3H10T1/2 cultures treated with Brefeldin A (5 *μ*M) up to 24 h. *β*-actin was used as a loading control. (**b**). Analysis of ability of adipose tissue macrophages to clear apoptotic adipocytes in *in vitro* co-culture system. Representative images and quantitative analysis of fluorescence intensity of green (DiO-macrophages) and red (BODIPY-lipid) are shown. (**c**) Quantitative analysis of fluorescence intensity of red (BODIPY-lipid) and green (DiO-macrophages) to compare adipocyte clearance rates between groups. Quantifications are representative of three individual experiments. (**d**) qPCR analysis of efferocytosis-related genes in adipose tissue macrophages co-cultured with dying or live adipocytes, and macrophages cultured without adipocytes (no AC) . Error bars indicated S.E.M. of three individual experiments (in comparison to GM + dying adipocytes controls, *P<0.05, **P<0.01, ***P<0.001)

To recapitulate *in vitro* adipocyte clearance by macrophages, a reconstructed *in vitro* model of adipose tissue macrophages and apoptotic adipocytes was used.^15^ F4/80+ macrophages were isolated by magnetic cell sorting (MACS) from gWAT of C57BL/6 mice. Differentiated adipocytes from C3H10T1/2 cells were treated with Brefeldin A to induce apoptosis for 24 h. As shown in Figure 2a, adipocytes underwent apoptosis indicated by increased levels of cleaved caspase-3 and decrease in perilipin 1 (Plin1) expression over the course of Brefeldin A treatment (Figure 2a). Therefore, we used differentiated adipocytes treated with Brefeldin A for 24 h as apoptotic adipocytes for co-culture. Macrophages were labeled with a traceable lipophilic dye, DiO, and lipids in adipocytes were labeled with fluorescent fatty acid analog BODIPYC12. Adipocyte clearance was monitored by the loss of BODIPY fluorescence intensity using long-term live imaging. In addition, pro-inflammatory M1-like and anti-inflammatory M2-like phenotypes were induced by 24 h treatment with lipopolysaccharide (LPS) and interleukin 4 (IL4), respectively. The expression levels of several pro- and anti-inflammatory genes were confirmed in macrophages by quantitative PCR (qPCR) (Supplementary Figure S1). In addition to the gene expression levels, macrophages treated with LPS and IL4 demonstrated morphological characteristics of M1- and M2-polarized macrophages, respectively. ^18^ As shown in Figure 2b, IL4-induced M2-like macrophages cleared the adipocytes more rapidly compared to LPS-treated macrophages, with a half-life of 4 *versus* 8 h, respectively, suggesting efficient anti-inflammatory removal of dying adipocytes by M2-like macrophages (Figures 2b and c). Next, we analyzed the gene expression levels at 4 h after co-culture to capture gene expression levels during active efferocytosis. Consistent with previous reports,^15^ Alox15 expression in macrophages was upregulated by co-culture with dying adipocytes, as shown in Figure 2d. We also examined genes involved in efferocytosis and found that the macrophage scavenger receptor 1 (MSR1) and a phosphatidyserine receptor,^19^ CD300 antigen-like family member F (CD300LF) were upregulated in macrophages co-cultured with dying adipocytes (Figure 2d), indicating involvement of efferocytosis.

**Figure 3:**
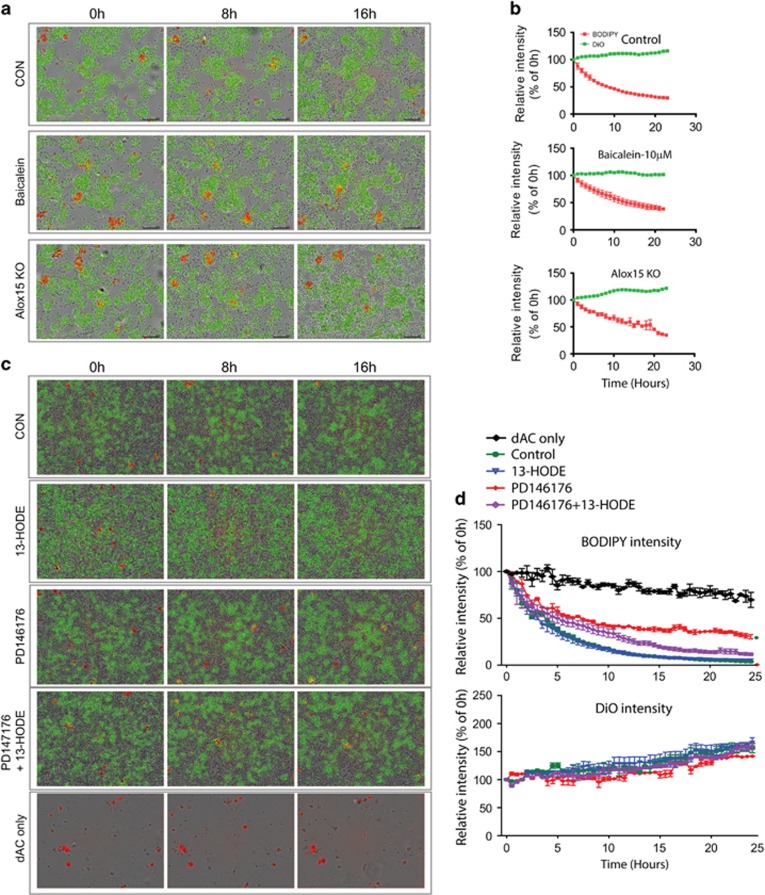
Effects of genetic deletion and chemical inhibition of Alox15 on adipocyte clearance by macrophages. Comparison of phagocytic ability of adipose tissue macrophages to clear dying adipocytes in *in vitro* co-culture system. (**a**,**b**) Adipose tissue macrophages were obtained from gWAT of Alox15 KO or WT mice (control, baicalein). A total of 10 *μ*M of baicalein (Bai), or vehicle were treated during long-term imaging. Representative images (**a**) and quantitative analysis (**b**) of fluorescence intensity of green (DiO-macrophages) and red (BODIPY-lipid) are shown. (**c**,**d**) Adipose tissue macrophages were obtained from gWAT of WT mice, and 10 *μ*M of PD146176 or 68 *μ*M of 13-HODE were treated during long-term imaging. Representative images (**c**) and quantitative anlaysis (**d**) of fluorescence intensity of green (DiO-macrophages) and red (BODIPY-lipid) are shown. Error bars indicated S.E.M. of three individual experiments per condition

Pharmacological inhibition and genetic deletion of Alox15 were used to determine whether Alox15 activity is required for efferocytosis of apoptotic adipocytes. Adipose tissue macrophages were obtained from gWAT from C57BL/6 and treated with baicalein^20^ (Figures 3a and b) or PD146176 (Figures 3c and d), pharmacological inhibitors of Alox15.^21^ For genetic deletion of Alox15, adipose tissue macrophages were obtained from gWAT of Alox15 KO mice. Long-term live imaging analyses indicated that inhibition of Alox15 activity by baicalein and deletion of Alox15 gene expression significantly delayed lipid clearance by macrophages with half-lives of 8.1  and 19.1 h, respectively, compared to control conditions with a half-life of 4.9 h (Figures 3a and b; Supplementary Figure S2). In addition, significant inhibitory effects of PD146176 on lipid clearance by macrophages were observed, showing 69.7±2.4% clearance with 10 *μ*M PD146176, compared to 94.6±0.7% under M2 control conditions at 24 h (Figures 3c and d). Next, we hypothesized that Alox15 lipid metabolites increased the phagocytic ability of macrophages to clear dying adipocytes. To test this, M2 macrophages co-cultured with dying adipocytes were treated with 13-HODE. Although 13-HODE did not significantly increase the adipocyte clearance rate compared to that in M2 macrophage controls, it reversed the inhibitory effect of the Alox15 inhibitor, PD146176, on phagocytosis by macrophages, recovering the lipid clearance from 69.7±2.4 to 88±1.3%. To confirm the macrophage-specific effects on lipid clearance, we measured red fluorescence intensity in the absence of macrophages. Although the data indicated a gradual decrease in red fluorescence, the levels remained relatively constant, showing a 30% decrease at 24 h compared to that at 0 h, indicating that macrophages actively cleared lipid. These data suggested that expression and activity of Alox15 is required for efferocytosis of apoptotic adipocytes by adipose tissue macrophages.

### Alox15 is required for efferocytosis of dying adipocytes by macrophages *in vivo*

Next, we used Alox15 KO mice to investigate the functional roles of Alox15 expression in adipose tissue remodeling. As mentioned above, it was previously reported that adipose tissue remodeling by *β*3-adrenergic stimulation required non-inflammatory removal of dying adipocytes by macrophages to support proliferation and differentiation of adipocyte progenitors,^6, 22^ which served as adipogenic niches.^22^ Thus, it was hypothesized that if Alox15 expression is required for the clearance of adipocyte death, then remodeling of adipose tissue by *β*3-adrenergic stimulation should be impaired in Alox15 KO mice.

To test this, mice were treated with CL for up to 5 consecutive days and were injected with BrdU on day 3 of CL treatment—the peak time of cell proliferation.^23^ Adipose tissues were examined at 4 h and at 2 days after BrdU injection.

**Figure 4:**
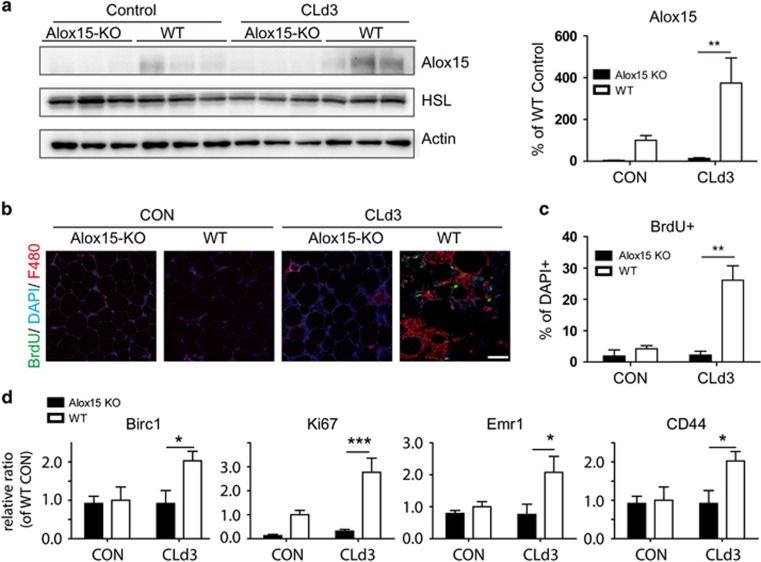
Alox15 is required for macrophage recruitment and cell proliferation in remodeling of gWAT induced by *β*3-adrenergic receptor stimulation. (**a**) Immunoblot analysis and quantification of Alox15 expression in gWAT from WT mice and Alox15 KO mice treated with CL for 3 days and untreated controls. *β*-actin was used as a loading control. (**b**) Representative images of paraffin sections of gWAT of mice treated with CL for 3 days and untreated controls, stained with BrdU and F4/80. Nuclei were counterstained with DAPI. Bars=20 *μ*m. (**c**). Quantification of BrdU+ cells. (**d**). qPCR analysis of genes related to proliferation and macrophage markers in gWAT from WT mice and Alox15 KO mice treated with CL for 3 days and untreated controls. Error bars indicated S.E.M. of 3 individual experiments per condition. *P<0.05, **P<0.01, ***P<0.001

The levels of proliferation and macrophage recruitment were determined by qPCR and histological examination on day 3 after CL treatment. It was confirmed that protein levels of Alox15 increased after 3 days of CL treatment in gWAT of WT mice, whereas Alox15 KO mouse tissues showed absence of Alox15 expression (Figure 4a). An adipocyte marker highly expressed in differentiated adipocytes, hormone-sensitive lipase (HSL), was expressed at a similar level in both WT and Alox15 KO mice (Figure 4a). Histological analysis of F4/80+ cells indicated that macrophage recruitment by *β*3-adrenergic stimulation was absent in gWAT of KO mice, whereas gWAT of WT mice recruited macrophages forming CLS (Figure 4b). BrdU staining of histological sections demonstrated that cell proliferation was also reduced in KO mouse (Figures 4b and c). Consistently, the expression levels of genes (Birc1, Ki67) involved in proliferation did not increase in the KO mouse (Figure 4d). Gene expression levels of macrophage markers were also examined, revealing that Emr1 (gene for F4/80) and CD44 expression levels were not increased in response to *β*3-adrenergic stimulation in gWAT of the KO mouse (Figure 4d).

**Figure 5:**
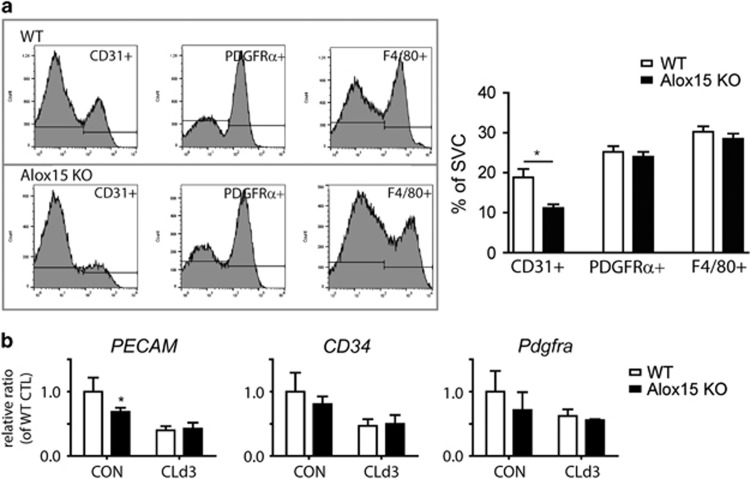
Analysis of markers of adipocyte progenitors and endothelial cells in gWAT of Alox15 KO mice. (**a**) Representative flow profiles and quantification of CD31+, PDGFR*α*+ and F4/80+ cells in SVC fraction from gWAT of WT and Alox15 KO mice. Error bars indicated S.E.M. of four individual experiments per condition (*P<0.05). (**b**) qPCR analyses of endothelial cell (*PECAM*), stem cell (*CD34*), and adipocyte progenitor (*Pdgfra*) markers in gWAT of WT and Alox15 KO mice. Error bars indicated S.E.M. of three individual experiments per condition (*P<0.05)

Cell proliferation may be affected by the distribution of the progenitor population; therefore, markers of endothelial cells (CD31), macrophages (F4/80), and adipocyte progenitors (platelet-derived growth factor receptor alpha (PDGFR*α*)) in the stromal vascular cell (SVC) fraction of gWAT were analyzed by fluorescence-activated cell sorting. CD31+ cells were significantly lower in Alox15 KO mice than that in controls, whereas PDGFR*α*+ progenitors and F480+ macrophages were present at similar levels in KO and WT mice (Figure 5a). In addition, qPCR analyses demonstrated slightly lower basal expression levels in the endothelial cell marker, platelet endothelial cell adhesion molecule (Pecam, also called CD31) in gWAT of Alox15 KO mice compared to WT mice. Consistent with the flow cytometry data, the gene expression levels of a common stem cell maker (CD34), and Pdgfra in gWAT were similar between genotypes and treatments (Figure 5b).

**Figure 6:**
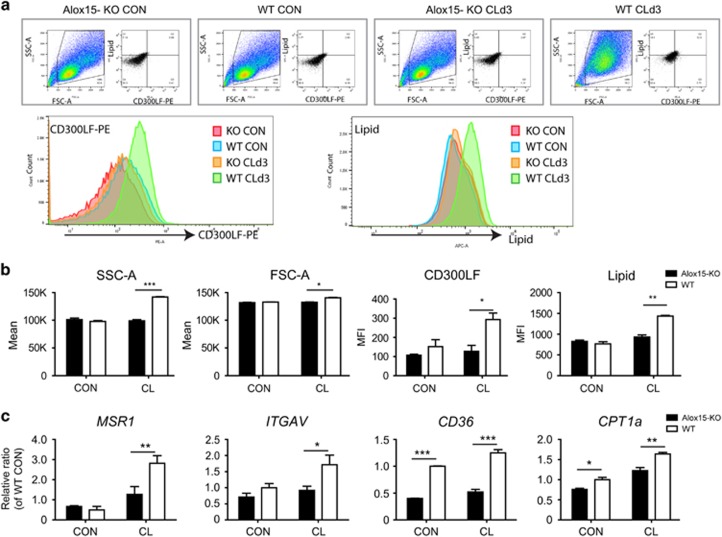
Alox15 is required for efferocytosis in remodeling of gWAT induced by *β*3-adrenergic receptor stimulation. (**a**) Flow cytometric analysis of CD300LF and lipid accumulation in macrophages isolated from WT and Alox15 KO mice treated with CL for 3 days and untreated controls. Representative flow profiles of two individual experiments per condition are shown. (**b**) Quantification of SSC-A, FSC-A, and mean fluorescence intensity of CD300LF-PE and LipidTox Deep Red. Error bars indicated ranges of two individual experiments as biological replicates of pooled tissue from three mice. (**c**) qPCR in macrophages of gWAT of WT and Alox15 KO mice treated with CL for 3 days and untreated controls. Error bars indicated S.E.M. of three individual experiments per condition. *P<0.05, **P<0.01, ***P<0.001

To evaluate levels of efferocytosis in macrophages from gWAT of Alox15 KO mice, CD300LF expression and lipid accumulation in macrophages were measured by flow cytometry. As an indication of lipid engulfment from dying adipocytes, macrophages isolated from gWAT of CL-treated WT mice showed increased lipid accumulation. Moreover, the expression of the phosphatidylserine receptor^19^ CD300LF was upregulated in macrophages from gWAT from CL-treated mice (Figures 6a and b). In contrast, this phenomenon was not observed in KO mice treated with CL (Figures 6a and b). In addition, macrophages from gWAT of CL-treated WT mice increased cell size and complexity, indicated by forward scatter area (FSC-A) and side scatter area (SSC-A) analyses (Figures 6a and b). To gain insight into the pathways underlying the defects in the efferocytosis of Alox15 KO macrophages, gene expression related to efferocytosis in macrophages isolated from gWAT of control and CL-treated WT and KO mice were examined. Expression levels of *MSR1* and integrin alpha V (*ITGAV*),^24^ genes involved in efferocytosis, were upregulated by CL treatment in macrophages from gWAT of WT mice, but not in macrophages of Alox15 KO gWAT (Figure 6c). As described above, Alox15 can generate peroxisome proliferator-activated receptor gamma (PPAR*γ*) ligands, including 13-HODE and 9-HODE in macrophages after CL treatment,^15^ and important roles of PPAR*γ* were established in efferocytosis.^25^ Especially, cluster of differentiation 36 (CD36), a PPAR*γ* target gene involved in lipid uptake and efferocytosis, was significantly downregulated in macrophages from KO mice. Carnitine palmitoyltransferase Ia (CPT1a) were significantly lower in macrophages isolated from KO mice compared to WT mice (Figure 6c), implying that Alox15 expression in macrophages is responsible for lipid metabolism required for the efficient efferocytosis of dying adipocytes.

### Alox15 is required for differentiation of adipocyte progenitors in *β*3-adrenergic stimulation-induced adipose tissue remodeling

**Figure 7:**
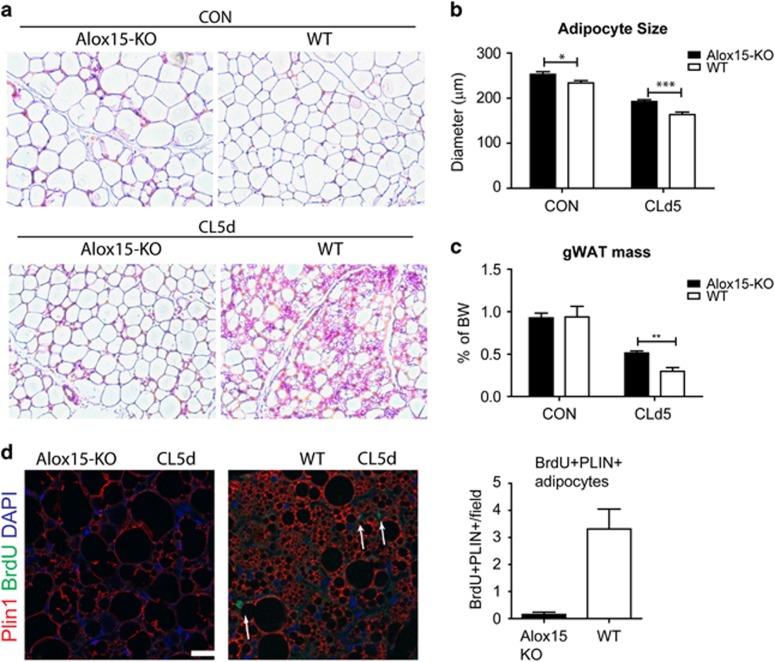
Alox15 is required for *de novo* adipogenesis in remodeling of gWAT induced by *β*3-adrenergic receptor stimulation. Representative images of H/E staining of paraffin sections of gWAT from WT and Alox15 KO mice treated with CL for 5 days or untreated controls. (**b**,**c**) Analyses of adipocyte size (**b**) and tissue weight (**c**) of gWAT from WT and Alox15 Ko mice treated with CL for 5 days or untreated control. Error bars indicated S.E.M. of four individual experiments per condition. (**d**) Representative images and quantification of BrdU and Perilipin 1 (PLIN1) staining in paraffin sections of gWAT from WT and Alox15 KO mice treated with CL for 5 days. Arrows indicate BrdU+Plin1+ adipocytes. Nuclei were counterstained with DAPI. Bars=20 *μ*m

Next, the levels of newly generated adipocytes were examined during adipose tissue remodeling by *β*3-adrenergic stimulation in Alox15 KO mice. As mentioned above, mice were treated with BrdU at day 3 of CL treatment, and BrdU-labeled proliferating cells were traced by day 5 of CL treatment. Hematoxylin/eosin (H/E) staining indicated appearance of multilocular adipocytes in gWAT of WT mice after 5 days of CL treatment, but fewer in CL-treated KO mice (Figure 7a). Furthermore, histological examination indicated that adipocytes were larger in KO mice than in WT mice under control conditions (Figure 7b). Although CL treatment reduced adipocyte size and adipose tissue mass in both genotypes, the extents of these catabolic responses were reduced in gWAT of KO mice compared to that in WT mice (Figures 7b and c). Consistently, expression levels of Pparg were lower in gWAT of Alox15 KO mice compared to WT mice, whereas other adipocyte markers (CCAAT/enhancer-binding protein alpha, *Plin1*, *Lipe*, and *Leptin*) were not differentially regulated in Alox15 KO mice (Supplementary Figure S3). As expected from previous observations, BrdU+ Plin1+ small adipocytes were detected by immunohistochemical analysis of gWAT of WT mice treated with CL for 5 days (Figure 7d) (i.e., BrdU+ Plin1+ adipocytes indicates new multilocular adipocytes from proliferating progenitors that incorporated BrdU 2 days earlier in gWAT of WT mice). In contrast, BrdU+ new adipocytes were almost undetectable in gWAT from KO mice (Figure 7b), which is comparable to untreated control conditions, indicating cellular restoration did not take place in gWAT of Alox15 KO mice. Collectively, the data suggested that Alox15 is required for macrophage recruitment and cell proliferation, which is required for *de novo* adipogenesis as restorative remodeling of the adipose tissue.

## Discussion

With the recognition of the central roles of adipose tissue in the regulation of metabolic homeostasis and in the pathogenesis of metabolic diseases, strategies targeting adipose tissue for therapeutic purpose have emerged. Although adipocytes are major parenchymal cells in the adipose tissue, complex mixtures of other cell types^26^ render highly plastic characteristics to adipose tissues in various physiological and pathological conditions.^4^ In this regard, understanding cellular and molecular players in *in vivo* adipose tissue remodeling is critical to harness adipose tissue for therapeutic purpose.

Apparently, adipose tissue remodels through adipocyte death, clearance of dying/dead adipocytes and *de novo* generation of adipocytes from progenitors, and contributes to homeostatic turnover and adaptation to metabolic challenges.^5^ However, the serial mechanistic events underlying adipocyte death and removal by tissue macrophages have not been fully described. In this regard, the present work demonstrates that adipose tissue remodeling by *β*3-adrenergic receptor stimulation involves non-inflammatory clearance of apoptotic fat cells by tissue macrophages that express high levels of Alox15. *In vivo* genetic deletion of Alox15 and *in vitro* reconstruction systems demonstrated that Alox15 expression in macrophages was required for efficient efferocytosis of dying adipocytes by macrophages, adipogenic differentiation of progenitors, and restorative remodeling of adipose tissue.

Although tissue-resident macrophages exist in almost every organ,^27^ elegant studies suggested specialized functions of adipose tissue macrophages sensing metabolic challenges in a lipid-rich environment.^10^ In particular, obese condition increases the frequency of pro-inflammatory macrophages in adipose tissues, resulting in M2–M1 shift in polarization status.^28, 29^ Furthermore, macrophages are often detected as multinucleated giant cells in CLS,^9, 11, 12^ which is an indication of unresolved inflammation and a major source of pro-inflammatory cytokines,^11^ which appears to contribute to the pathogenesis of insulin resistance and chronic metabolic disease.^29^ Although distinct distributions of M1 and M2 macrophages in adipose tissues of various physiological and pathological conditions have been reported,^30^ the phagocytic ability of each phenotype to clear dying adipocytes has not been characterized. Importantly, the present study demonstrated that IL4-stimulated M2-like macrophages possess higher capacity to clear dying adipocytes than LPS-treated M1-like macrophages. Presumably, the clearance of dying adipocytes is distinct from other cell types, especially when considering the large size and high triglyceride contents of adipocytes. Thus, rapid disposal or consumption of lipids in macrophages will be required for efficient efferocytosis and prevention of an inflammatory response. Supporting this idea, we demonstrated that metabolic programs in M2 macrophages are apt to handle lipids as they possess higher capacity for lipid uptake and free fatty acid oxidation, a function that is suppressed in M1 polarized macrophages.^31^ Furthermore, delayed removal of dying adipocytes by M1 macrophages can explain the abundance of unresolved adipocyte death and inflammatory response of M1 macrophages in hypertrophied adipose tissues. In this regard, promoting efferocytosis of apoptotic adipocytes can be utilized as a therapeutic target to prevent chronic inflammation and development of insulin-resistant states in adipose tissue.

PPAR*γ* is a part of the nuclear hormone receptor family and a well-known master regulator in adipogenesis and lipid metabolism.^25^ Transcriptional activity of PPAR*γ* in macrophages can support anti-inflammatory roles by modulating cytokine production, inducing alternative activation programs^32^ and promoting uptake of apoptotic cells.^25^ Particularly, for efferocytosis, PPAR*γ* expression is necessary for the expression of CD36,^33^ a major scavenger receptor. In the current study, distinct phenotypes of macrophages from the adipose tissue of Alox15 mouse were identified, which were characterized by decreased levels of PPAR*γ* target genes, including CD36 and CPT1a, that are required for free fatty acid uptake and mitochondrial *β*-oxidation. 13-HODE treatment partially reversed the inhibitory effect of baicalein on phagocytosis of dying adipocyte. This indicates that Alox15-dependent PPAR*γ* ligands^15^ are important for efficient efferocytosis of dying adipocytes; however, the roles of other metabolites or function of Alox15 cannot be excluded.

Macrophages are an important source for lipid-signaling molecules, and regulation of lipid-modifying enzymes in macrophages can orchestrate inflammation and resolution.^13, 30, 31, 34, 35^ For instance, lipoxygenase can oxidize eicosanoids and polyunsaturated fatty acids, generating pro- and anti-inflammatory signaling molecules. Of particular interest, Alox15 has been implicated in the generation of specialized proresolving mediators such as lipoxin and resolvin biosynthesis.^13^ These mediators stimulate the clearance of apoptotic cells and debris, having fundamental roles in inflammation-resolution.^35^ It is possible that Alox15 expression engages in the generation of proresolving lipid species to potentiate efferocytosis process in *β*3-adrenergic remodeling of adipose tissues. Recently, Gpr132, a receptor for 9-HODE has been reported as a molecular player in efferocytosis,^36^ suggesting potential involvement of Alox15 metabolites in efferocytosis. Further studies on lipid metabolite profiles during adipose tissue remodeling will provide insight into the molecular mechanisms of the Alox15 pathway in the efferocytosis of apoptotic adipocytes.

Although substantial evidence supports important roles of Alox15 activity in resolution and anti-inflammatory pathways, pro-inflammatory/pathogenic roles of Alox15 in adipose tissue have been reported.^37, 38^ For example, upregulation of Alox15 in visceral adipose tissue is implicated in the development of insulin resistance.^17^ Genetic deletion of Alox15 has been shown to be protective against diet-induced obesity-related metabolic abnormalities in mouse models.^39^ Similarly, several studies indicated that upregulation of Alox15 expression during high-fat feeding is responsible for the production of pro-inflammatory cytokines and insulin resistance.^40^ However, in this study, we found that the pro-inflammatory Alox15 products 12-HETE and 15-HETE, were not upregulated after CL treatment, suggesting that the pro-inflammatory role of Alox15 is induced by high-fat diet feeding, but not by *β*-adrenergic stimulation. Thus, key factors that may regulate this dual role of Alox15 include diet,^41^ and lipid metabolites generated by the enzyme.^37^ Further investigation of Alox15 function in adipose tissue biology is needed in the context of adipose tissue remodeling, including chronic effects of defects in efferocytosis of apoptotic adipocytes.

Although non-inflammatory removal of dying adipocytes is crucial for maintaining metabolic homeostasis, molecular and cellular players in efferocytosis of dying adipocytes are poorly understood. In addition, obesity-related metabolic disease has been viewed as unresolved inflammatory disease; thus, regulation of immunity is a promising approach for treating chronic metabolic disorders.^35, 42^ In the present study, using a combination of an *in vivo* genetic deletion model and an *in vitro* reconstruction system, pivotal roles were identified for Alox15 expression in macrophages for non-inflammatory efferocytosis of dying adipocytes and *β*3-adrenergic remodeling of adipose tissue. Controlling efferocytosis of apoptotic adipocytes by macrophages opens up an attractive therapeutic opportunity to ameliorate obesity-related metabolic diseases.

## Materials and Methods

### Animals

All animal protocols were approved by the Institutional Animal Care and Use Committees at Yonsei University. Alox15 KO mice (JAX Mice Stock # 002778, Bar Harbor, ME, USA) were purchased and bred as described.^43^ C57BL/6 mice (5–6-weeks old, male) were purchased from Orient Bio (Gyeonggi-Do, South Korea) and were fed a standard chow diet. For ADRB3 stimulation, mice were injected daily with CL316.243 (Sigma, St. Louis, MO, USA) (1 mg/kg/day, intraperitoneal injection) for up to 5 days.

### SVC fractionation and flow cytometry

SVC and adipocyte fractions were isolated from mouse gWAT as previously described.^23^ Fractionated adipoctyes were used for the mRNA analysis. For flow cytometric analysis, SVC were processed for cell-surface marker staining using anti-F4/80- conjugated with phycoerythrin PE/Cy7, anti-CD31- conjugated with fluorescein isothiocyanate (FITC) and anti-PDGFR*α* conjugated with phycoerythrin (PE), (rat, 1:200, Biolegend, San Diego, CA, USA). HCS LipidTOX Deep Red Neutral Lipid Stain H34477 (Thermo Fisher Scientific, Waltham, MA, USA) was used for neutral lipid staining. BD FACSAria III (BD Biosciences, San Jose, CA, USA) flow cytometer was used for flow cytometric analysis. Raw data were processed using FlowJo software (Tree Star, Ashland, OR, USA). F4/80+ macrophages were isolated by MACS using FITC conjugated anti-mouse F4/80 antibody (BioLegend, San Diego, CA, USA) and anti-FITC-magnetic beads (Miltenyi, Bergisch Gladbach, Germany) according to manufacturer's instruction.

### Lipidomics analysis

MACS-isolated F4/80+macrophages were homogenized by probe sonication on ice (3 × 10 s). The homogenates were supplemented with a mixture of internal standards, extracted, and the lipid extracts were subjected to LC–MS-based lipidomic analysis to determine fatty acyl lipidome according to the standard method described previously.^15^

### Cell cultures

C3H10T1/2 mouse embryonic fibroblasts (ATCC, Manassas, VA, USA ) were cultured to confluence in growth medium (DMEM supplemented with 10% fetal bovine serum (ThermoFisher Scientific) and 1% penicillin/streptomycin (ThermoFisher Scientific) at 37°C in a humidified atmosphere with 5% CO_2_ and then exposed to bone morphogenetic protein 4 (20 ng/ml, R&D system, Minneapolis, MN, USA) followed by exposure to differentiation medium (DMEM supplemented with 10% fetal bovine serum, 1% P/S, 2.5 mM isobutylmethylxanthine (Cayman Chemical, Ann Arbor, MI, USA), 1 *μ*M dexamethasone (Cayman Chemical), and 1 *μ*g/ml insulin (Sigma) for 3 days. For long-term culture, cells were maintained in medium containing 1 *μ*g/ml insulin for up to 10 d. MACS-isolated F4/80+ macrophages from gWAT were cultured at an initial concentration of 10^5^ cells/ml in growth medium. For the co-culture experiment, fully-differentiated adipocytes obtained from the C3H10T1/2 cells were treated with Brefeldin A (5 *μ*g/ml, BioLegend).^44^

For long-term imaging, differentiated C3H10T1/2 adipocytes were labeled with 4,4-difluoro-5-(2-thienyl)-4-bora-3a,4a-diaza-s-indacene-3-dodecanoic acid (BODIPY 558/568 C12) (ThermoFisher Scientific) and F4/80+ macrophages isolated from gWAT of mice were labeled with Vybrant DiO Cell-Labeling Solution (ThermoFisher Scientific, Waltham, MA USA) overnight. BODIPY-labeled C3H10T1/2 cells were detached, added to the DiO-labeled macrophages, and then co-cultured for 2 days.^15^ To induce M1 and M2 polarization, LPS (1 *μ*g/ml, Sigma) and IL4 (20 ng/ml, R&D system) were treated. Co-cultures were treated with pharmacological inhibitors of Alox15, baicalein (10 *μ*M, Sigma) or PD146176 (10 *μ*M, Tocris Bioscience, Bristol, UK) in the presence or absence of the Alox15 product 13-HODE (68uM, Cayman Chemical)^15^ for 2 days. To monitor the phagocytosis of the dying adipocytes by the macrophages, live cell imaging was performed every 30 min or 1h with IncuCyte ZOOM live cell imaging equipment, (ESSEN Bioscience, Ann Arbor, MI, USA), and the fluorescence intensity of the images was analyzed using IncuCyte ZOOM (ESSEN Bioscience) software.

### Gene expression analysis

RNA was extracted using TRIzol reagent (ThermoFisher Scientific) and converted into cDNA by using High Capacity cDNA synthesis kit (Applied Biosystems, Waltham, MA, USA). Overall, 50 ng of cDNA was analyzed in a 20 *μ*l qPCR reaction. Quantitative real time-PCR was performed using SYBR Green Master Mix (Applied Biosystems) and ABI StepOne PLUS (Applied Biosystems) for 45 cycles and the fold change for all the samples was calculated by the comparative cycle-threshold (Ct) method (i.e., 2−ΔΔCt method). Peptidylprolyl isomerase A was used as the housekeeping gene for mRNA expression analysis. cDNA was amplified using the following primers: MSR1 (Macrophage scavenger receptor 1) with 5′-GCTCACTTTGGACAAGGTACTG-3′ (forward) and 5′-GCTTAGTACTCCCCACTGGTT-3′ (reverse); Cd300lf (IREM-1) with 5′-CCCAGCAATCCAAGTACCCA-3′ (forward) and 5′-TCCAGAAACCCATCACCGAC-3′ (reverse); ITGAV with 5′-CGTCCTCCAGGATGTTTCTCC-3′ (forward) and 5′-TCACAGAGGCTCCAAACCAC-3′ (reverse). All other cDNAs were amplified using previously described primers.^15^ ^23^

### Western blot analysis

Protein extracts were prepared as previously described.^45^ Overall, 10 *μ*g of samples were electrophoresed and blotted to PVDF membrane (Bio-Rad, Hercules, CA, USA). Western blot was performed using primary antibodies against 15-lipoxygenase-1 (Mouse, Abcam, Cambridge, UK) caspase-3 (Rabbit, Cell Signaling, Danvers, MA, USA), cleaved form of caspase-3 (Rabbit, Cell Signaling), HSL (Rabbit, Cell signaling) and *β*-actin (Mouse, Santa Cruz Biotechnology, Dallas, TX, USA) and secondary anti-mouse and anti-rabbit horse-radish peroxidase antibodies (Cell Signaling Technology) as described previously.^45^ The blots were visualized with SuperSignal West Dura Substrate (ThermoFisher Scientific).

### Tissue processing and histology

gWAT was processed for histological sections, and 5-*μ*m-thick paraffin sections were subjected to H/E staining or immunohistochemical analysis, as previously described.^45^ The antibodies used for immunochemical detection were anti-15-lipoxygenase-1 antibody (Mouse, Abcam), cleaved caspase-3 (Rabbit, Cell Signaling), Perilipin A (Rabbit, Santa Cruz Biotechnology), BrdU-FITC (Roche Diagnostics, Mannheim, Germany) and anti-F4/80 antibody (Rat, AbD Serotec, Raleigh, NC, USA). The secondary antibodies used were goat anti-mouse-Alexa Fluor 488, goat anti-rabbit-Alexa Fluor 594 and goat anti-rat-Alexa Fluor 594 (ThermoFisher Scientific, Molecular Probes). The omission of primary antibody or normal rabbit, or mouse IgG controls (Santa Cruz Biotechnology) were used as a negative control. DAPI (Sigma) was used for nuclear counterstaining.

Cells were imaged on a Zeiss confocal laser-scanning microscope (LSM 710 META, Zeiss, Jena, Germany). Images were acquired following excitation with 405 nm laser diode, 488 nm Argon laser and 561 nm DPSS laser followed by filter set 49-DAPI, 10-GFP, and 20-Rhodamine for DAPI, Alexa Fluor 488, and Alexa Fluor 594, respectively, with a C-Apochromat 40XC/1.2 W corr.

BrdU+Plin1+ adipocytes were counted in at least five of 400 × fields from each mouse, and the mean of Plin1+BrdU+ +adipocytes/400 × field obtained from individual mice was used to compare the number of new adipocytes between WT and Alox15 KO mice. Adipocyte size was measured in paraffin sections stained for H/E, and was calculated as the mean diameter of at least 200 random adipocytes in four of 200 × fields per each sample. All quantification of histologic samples was carried out as blind analyses.

### Statistical analysis

Statistical analyses were performed with GraphPad Prism 5 software (GraphPad Software, La Jolla, CA, USA). Data are presented as mean±S.E.M. Statistical significance between two groups was determined by the unpaired *t*-test or the Mann–Whitney test, as appropriate. Comparison among multiple groups was performed using a two-way ANOVA, with Bonferroni *post hoc* tests to determine the relevant *P*-values. A half-life was calculated by non-linear regression analysis with one-phase association model.

## Abbreviations

Alox15: arachidonate 15-lipoxygenase
gWAT: gonadal white adipose tissue
KO: knockout
CLS: crown-like structure
WAT: white adipose tissue
CL: CL316,243
HODE: hydroxyoctadecadienoic acid
HETE: hydroxyeicosatetraenoic acid
MACS: magnetic cell sorting
Plin1: perillipin 1
LPS: lipopolysaccharide
IL4: interleukin 4
MSR1: macrophage scavenger receptor 1
CD300LF: CD300 antigen-like family member F
WT: wild type
HSL: hormone-sensitive lipase
PDGFR*α*: platelet-derived growth factor receptor alpha
SVC: stromal vascular cell
ITGAV: integrin alpha V
PPAR*γ*: peroxisome proliferator-activated receptor gamma
CD36: cluster of differentiation 36
CPT1a: carnitine palmitoyltransferase 1a
FITC: fluorescein isothiocyanate
BODIPYC12: 4,4-difluoro-5-(2-thienyl)-4-bora-3a,4a-diaza-s-indacene-3-dodecanoic acid
