# Supplementary material for: The contribution of arachidonate 15-lipoxygenase in tissue macrophages to adipose tissue remodeling
Source: Cell Death Dis. 2016 Jun 30;7(6):e2285–. doi: 10.1038/cddis.2016.190 (PMC5108340; doi:10.1038/cddis.2016.190)
Supplement: Supplementary Information [file cddis2016190x1.doc]

Supplemental data:


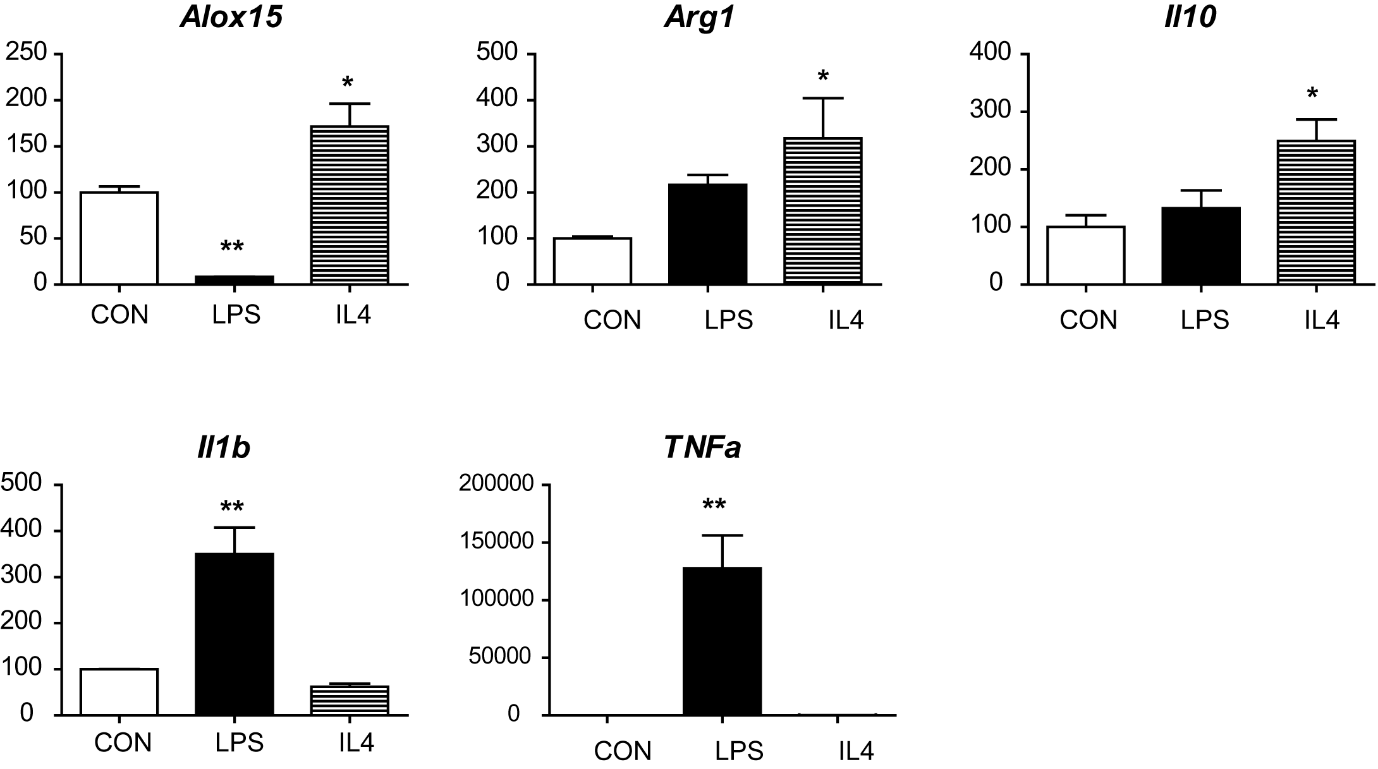


**Figure S1. qPCR analysis M1 and M2 marker expression in adipose tissue macrophages treated with LPS, IL4, or vehicle (CON)**


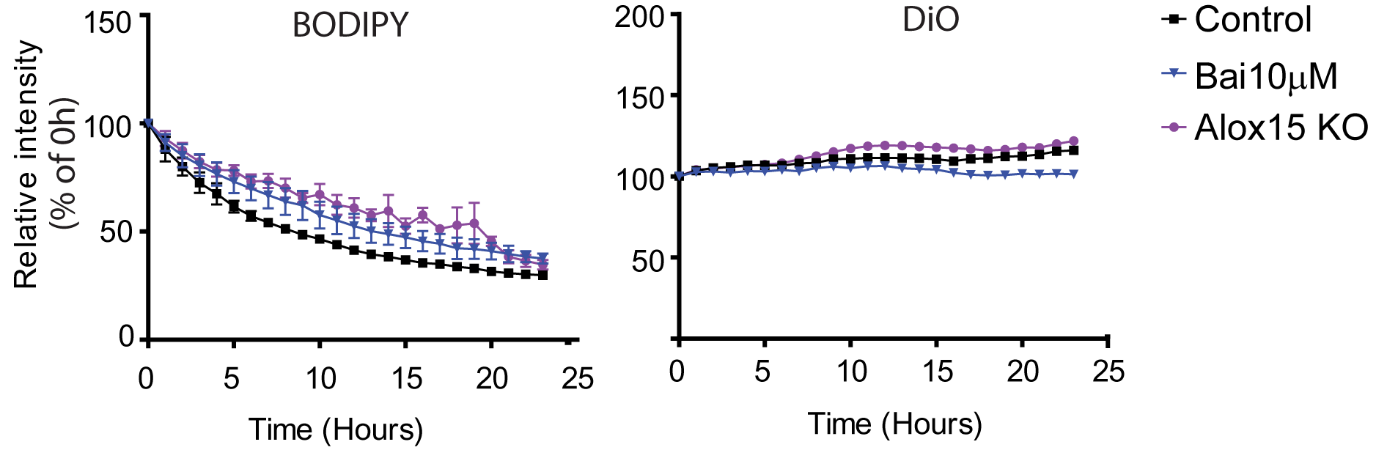


**Figure S2. Effects of pharmacological inhibition of Alox15 on adipocyte clearance by macrophages.**


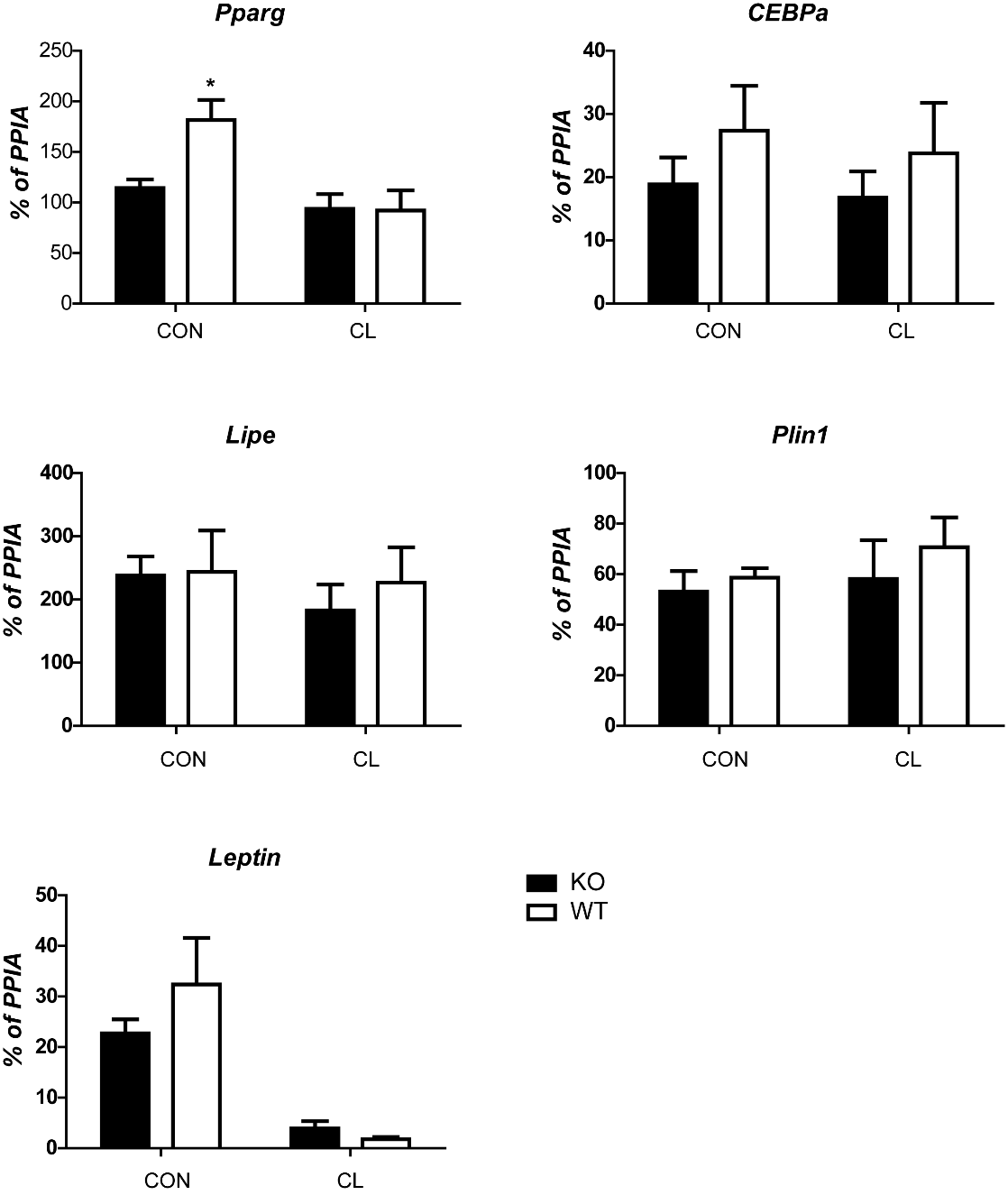


**Figure S3. qPCR analysis of adipogenic gene expression in gWAT of WT and Alox15 KO mice treated with CL for 3 days or untreated controls**
